# Comparison of flowmeter (transit time flow measurement) values for graft flow in three different surgical methods for isolated coronary artery bypass surgery

**DOI:** 10.5830/CVJA-2023-029

**Published:** 2023-07-04

**Authors:** Ferhat Borulu, Ümit Arslan, Eyüpserhat Çalik, Bilgehan Erkut

**Affiliations:** Cardiovascular Surgery Department, Faculty of Medicine,Ordu University, Ordu, Turkey; Cardivascular Surgery Department, Faculty of Medicine, Atatürk University, Erzurum, Turkey

**Keywords:** transit time flow meter, coronary artery bypass surgery

## Abstract

**Background:**

Graft patency is the most important factor in coronary artery bypass surgery. This study aimed to compare the relationship between three different surgical methods and transit time flow measurement (TTFM), which is used to detect technical problems in anastomoses performed during coronary artery bypass graft operations and to correct them if necessary.

**Methods:**

A total of 110 patients undergoing isolated coronary artery bypass surgery were analysed. Of these patients, 48 were operated on by inducing cardiopulmonary arrest (group 1), 33 were operated on without inducing cardiac arrest (group 2) during cardiopulmonary bypass surgery, and 29 underwent surgery on the off-pump beating heart (group 3). TTFMs were performed on all the patients’ grafts. Additional surgical intervention requirements, the need for intra-operative and postoperative inotropic support, and all postoperative follow-up data were compared.

**Results:**

In total, 110 patients were measured for 301 grafts. Due to insufficient measurements performed on these patients, additional surgical intervention was performed on five grafts in group 1, five grafts in group 2, and seven grafts in group 3. These interventions enabled a normal flow rate to be achieved. The number of grafts that required revision was highest in group 3. There was no difference between the groups in terms of demographic data, EuroSCORE II, preoperative ejection fraction, postoperative complications and mortality rate.

**Conclusion:**

TTFM is important for detecting technical problems in grafts. We believe that all surgical methods can be applied more safely by controlling graft flow.

Grafting with sufficient flow is one of the most important parameters in coronary artery bypass surgery. The absence of sufficient flow in the graft after a recently performed anastomosis can lead to significant increases in mortality rate by causing new infarctions during the procedure and reducing its benefits. Serious increases in mortality and morbidity rates can occur due to early graft failure in the postoperative period.^1^,^2^

If the flow problems that arise due to surgical techniques are detected and corrected with necessary interventions, the success of the procedure and the benefits patients can receive from surgery will increase. Nonetheless, research about which surgical methods are more beneficial in terms of anastomosis quality has remained controversial for years. This study aimed to compare anastomoses performed with three different methods during coronary artery bypass surgery with objective parameters that included transit time flow measurement (TTFM).

## Methods

This study included patients who had undergone isolated coronary artery bypass surgery by the same surgical team at the Cardiovascular Surgery Clinic of Ataturk University Faculty of Medicine between August 2017 and March 2019. The study was approved by the local ethics committee, and the patients were retrospectively analysed. At least 48 of these patients were operated on under cardiopulmonary bypass (CPB) by inducing cardiac arrest, 33 were operated on under CPB without inducing cardiac arrest, and 29 underwent off-pump beating heart without CPB.

The grafts in all patients were checked by measuring transit time flow at the end of the operations. Necessary graft revisions were performed for patients with problems in flow. All interventions and measurements were recorded. All the patients’ demographic data, EuroSCORE II data, operative data, and postoperative follow-up parameters were compared between the three groups.

TTFM is an effective method for intra-operatively evaluating graft patency. Although different series disagree about the sensitivity and specificity of TTFM,^3^,^4^ European myocardial revascularisation guidelines recommend using this method to control graft openings (Class I recommendation, level of evidence C).^5^,^6^ The device (TTFM) is compliant with the European Medical Device Directive 93/42/EEC and is also approved by the US Food and Drug Administration (FDA) [FDA 510(k) cleared no. K102595 and FDA 510(k) cleared no. K040228].

**Fig. 1:**
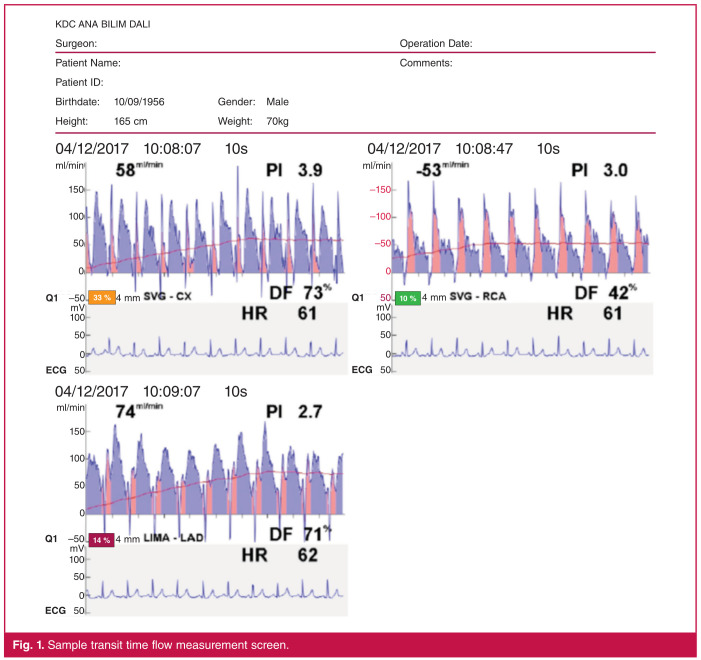
Sample transit time flow measurement screen.

The technique was developed to evaluate graft quality and to measure the blood flow through the graft with its special probe. The device our clinic used was the MediStim VQ-1101, MediStim ASA (Oslo, Norway). The measurements were performed after the anastomoses were completed for all grafts, and cardiac haemodynamic stabilisation was achieved in the patients. With this technique, the diastolic filling (DF), mean flow and pulsatility index (PI) can be measured (Fig. 1).

Based on the data from similar studies and recommendations from the manufacturer, we accepted the PI value as the main criterion for flow quality. We performed additional interventions on the grafts with a PI value of ≥ 5. The decision for additional interventions was made according to the values that were obtained prior to protamine infusion. All grafts were measured from at least two segments and were evaluated by averaging. Although the decision for additional interventions was made according to the pre-protamine data, intergroup comparisons were made according to the post-protamine data.

For surgery, the patients in all groups were anaesthetised with standardised anaesthetic agents at our clinic. The mediastinum was reached by performing standard incisions and median sternotomies in all patients. Due to the number of vessels that required surgery and the number of grafts that would need to be used, left internal mammary artery (LIMA) and saphenous vein grafts were prepared.

Coronary bypass was performed on all patients in groups 1 and 2 using standard CPB. Patients in group 3 underwent surgery on the beating heart without entering the pump. Ascending aorta cannulations and right atrial two-stage venous cannulations were performed on all patients to proceed with CPB. Roller pumps and membrane oxygenators were used. Cardiac arrest was induced with antegrade cardioplegia after cross-clamping for myocardial protection in CPB, and distal and proximal anastomoses were performed under cross-clamping. Myocardial protection was achieved by repeated cardioplegia every 20 minutes until the anastomoses were completed. Body temperatures were lowered to 30–32°C.

In group 2, the patients underwent ascending aorta cannulations and right atrial two-stage venous cannulations, but the anastomoses were performed without cross-clamping and cardioplegia. The Octopus was used in the anastomoses performed on the beating heart without CPB.

Proximal anastomoses were performed with side clamping in all patients. All distal anastomoses were performed with 7.0 prolene sutures, and all proximal anastomoses were performed with 5.0 prolene sutures.

When haemodynamic stabilisation was achieved after the completion of the anastomoses, TTFMs were performed. Mechanical factors, such as torsion, spasms and air were primarily eliminated in the grafts that showed low PI values according to the TTFMs. Papaverine and nitroglycerin were used to eliminate any possible spasms, especially in arterial grafts. Probes were sent from the grafts into the native vessels through incisions made just above the distal anastomoses in patients whose PI values did not improve after the interventions.

With the aid of the Octopus, the anastomoses were renewed on the beating heart in patients who were considered to have stenosis in the anastomosis line. The procedure was terminated in patients who did not achieve the desired improvements in PI values despite technical intervention since there were no obvious changes in the electrogram. All procedures were recorded.

## Statistical analysis

The Number Cruncher Statistical System 2007 (Kaysville, Utah, USA) program was used for the statistical analysis. In the assessment of the study data, the distribution of the data was evaluated with the Shapiro–Wilk test and descriptive statistical methods (mean, standard deviation, median, frequency, ratio, minimum and maximum). In the comparison of the quantitative data, an ANOVA test was used for three or more groups with normal distribution, and the Kruskal–Wallis test was used for the groups without normal distribution. Post hoc tests were used to determine the differences. A chi-squared test was used to analyse the qualitative data. Statistical significance was set at p < 0.01 and p < 0.05 levels.

## Results

The mean ages of the patients in all three groups were 62.02 ± 9.66, 61.67 ± 9.56 and 61.38 ± 8.97 years, respectively. There were no statistical differences between them. The patients’ other demographic data are presented in Table 1. There were no significant differences between the EuroSCORE II evaluations of the groups (group 1: 1.17 ± 0.56, group 2: 1.43 ± 1.08 and group 3: 1.38 ± 0.82).

**Table 1:** Demographic data of the patients

*Demographics*	*Group 1 (n = 48)*	*Group 2 (n=33) =*	*Group 3 (n=29)*	*p-value*
Age (years)	62.02 + 9.66	61.67 + 9.56	61.38 + 8.97	0.958
EuroSCORE II	1.17 + 0.56	1.43 + 1.08	1.38 + 0.82	0.300
Gender, n (%)				0.283
Female	(29.2)	9 (27.3)	(13.8)	
Male	34 (70.8)	(72.7)	25 (86.2)	
COPD, n (%)	(16.7)	(12.1)	(13.8)	0.842
CRF, n (%)	(2.1)	(0.0)	(0.0)	0.521
Hypertension, n (%)	(58.3)	7 (51.5)	18 (62.1)	0.690
Hyperlipidaemia, n (%)	10(20.8)	(12.1)	(10.3)	0.382
Diabetes mellitus, n (%)	17 (35.4)	16 (48.5)	14 (48.3)	0.395
PAD, n (%)	(6.2)	(12.1)	(10.3)	0.641
LVEF (%)	53.48 + 7.7	50.94 + 10.27	52.34 + 7.31	0.416

COPD: chronic obstructive pulmonary disease, CRF: chronic renal failure,

PAD: peripheral arterial disease, LVEF: left ventricular ejection fraction.

On average, 3.19 ± 0.84, 2.79 ± 0.74 and 2.34 ± 0.9 grafts were used in the patients in groups 1 to 3, respectively. The left internal thoracic artery was used for the left anterior descending (LAD) artery in all patients (Table 2). The number of grafts used in the group undergoing cardiac arrest (group 1) under cardiopulmonary bypass was higher than in the other groups (p < 0.01). There were no significant differences between the groups in terms of intubation times, intensive care unit stay, service follow-up times and postoperative complications (Table 3). Although discharge times were found to be shorter in the patients undergoing surgery on the beating heart, these findings were not statistically significant.

**Table 2:** Number and locations of grafts used and technical problems

*Variables*	*Group 1 (n = =48)*	*Group 2 (n = 33)*	*Group 3 (n = 29)*
Total number of grafts, n	153	92	68
LIMA-LAD anastomosis, n (%)	48 (100)	33 (100)	(100)
Ao-Cx anastomosis, n (%)	39 (81.2)	27 (81.8)	15 (51.7)
Ao-RCA anastomosis, n (%)	36 (75.0)	23 (69.6)	14 (48.2)
Ao-DIA anastomosis, n (%)	30 (62.5)	9 (27.2)	10 (34.4)
Curl in the grafts, n (%)	2 (1.3)	2 (2.17)	2 (2.94)
Vasospasm, n (%)	2 (1.3)	2 (2.17)	2 (2.94)
Anastomosis problem, n (%)	1 (0.65)	1 (1.08)	2 (2.94)

LIMA: left internal mammary artery, LAD: left anterior descending artery, Ao:

aorta, Cx: circumflex artery, RCA: right coronary artery, DIA: diagonal artery.

**Table 3:** Operative and postoperative data of patients

*Variables*	*Group 1 (n = 48)*	*Group 2 (n=33)*	*Group 3 (n = 29)*	*p-value*
Postoperative complication, n (%)	14 (29.2)	8 (24.2)	12 (41.4)	0.326
Intra-aortic balloon pump, n (%)	0 (0.0)	1 (3.0)	(0.0)	0.308
Positive inotropic support, n (%)	9 (18.7)	8 (24.2)	5 (17.2)	0.757
Number of grafts used, n (%)	3.19 + 0.84	2.79 + 0.74	2.34 + 0.9	0.001*
Duration of intensive care (h)	49.44 + 11.72	60.27 + 21.64	61.79 + 28.25	0.096
Postoperative hospital stay (day)	5.54 + 1.65	5.52 + 0.87	5.83 + 1.51	0.431
Operation time (min) *Kruskal-Wallis test.	3.96 + 0.68	4.03 + 0.73	4.22 + 0.62	0.161

*Kruskal–Wallis test.

In total, 110 patients were measured for 313 grafts. Because LIMA grafts were used in all patients, the three groups were correctly compared in terms of the most important anastomoses. In the first measurement, the mean PI value was measured as 3.1 in 100 (90.9%) of 110 patients with LIMA grafts (PI ≤ 5). The flow was weak (PI ≥ 5) in five grafts (3.26%) in group 1, five grafts (5.43%) in group 2, and six grafts (8.82%) in group 3.

Table 4 presents the measurements and problems detected in the grafts. First, the presence of any torsion in the grafts was checked. No additional interventions were required after changes in the position of the saphenous veins resulted in significant improvements to the PI values of two grafts in group 1. In patients with PI values > 5 in the LIMA–LAD anastomoses measurements, pre-anastomosis administration was performed, and local papaverine and nitroglycerin were administered on the LIMA grafts. The PI values increased to normal limits in two of the patients in group 1 with this administration. The administration also led to the renewal of the anastomosis and improvement in the PI values of one patient. Moreover, PI values increased with local papaverine administration and position changes in two patients from group 2.

**Table 4:** Transit time flow measurement values

*Variable LIMA-LAD*	*p-value*	*Ao-Cx*	*p-value*	*Ao-RCA*	*p-value*	*Ao-DIA*	p-value
PI							
Group 1 3.03 + 1.54	0.001	3.71 + 1.87	0.438	3.35 + 2.05	0.313	2.45 + 0.81	0.001
Group 2 3.91 + 0.98		3.72 + 1.3		4.94 + 6.38		4.01 + 0.68	
Group 3 3.95 + 0.77		3.83 + 0.83		4.01 + 0.91		3.94 + 0.77	
HR							
Group 59.92 + 30.04	0.461	52.46 + 24.27	0.857	57.78 + 27.63	0.880	62.57 + 33.59	0.980
Group 255.18 + 20.39		51.15 + 13.36		54.87 + 17.48		59.67 + 19.93	
Group 363.31 + :23.83		56 + 21.38		57.93 + 18		57.1 + 20.46	
DF							
Group 1 72.6 + 7.54	0.403 66.59	+ 10.91	0.013	57.69 + 13.71	0.021	68.27 + 12.35	0.992
Group 2 71.91 + 8.68		59.48 + 10.04		64.96 + 7.73		67.78 + 5.7	
Group 369.72 + 11.76		68 + 8.28		65.86 + 9.42		68 + 10.02	

PI: pulsatility index, HR: heart rate, DF: diastolic filling, LIMA: left internal mammary

artery, LAD: left anterior descending artery, Ao: aorta, Cx: circumflex artery, RCA: right

coronary artery, DIA: diagonal artery.

The circumflex (Cx) anastomosis was renewed in one patient in group 2. In group 3, saphenous distal anastomoses were renewed in two grafts. Position changes proved sufficient for two grafts. For two patients, flow was increased through the use of locally administered drugs. Anastomosis revisions were performed with the support of the Octopus on the beating hearts of all patients. Although TTFMs were low in the saphenous vein grafts of group 3, none of these patients experienced significant electrocardiogram changes or haemodynamic problems.

In only one of the patients (group 1), a percutaneous intraaortic balloon pump was placed at the end of the CPB when the blood pressure was not sufficient despite inotropic support. There were no significant differences between groups 1 and 2 in terms of inotropic support that was initiated postoperatively. There was significantly less inotropic support in group 3 (p < 0.05). Although the mean number of grafts that was used in the patients who underwent operation on the beating heart was lower than in the other two groups, this difference was not statistically significant.

## Discussion

Aortocoronary bypass graft operations have been successfully performed to treat severe diseases and conditions of the coronary artery for many years. As in all surgeries, improvements have been made to coronary bypass surgery over time. However, discussions on which methods result in the most successful anastomoses and surgeries are ongoing. Among coronary artery bypass surgeries, anastomosis quality is considered to be a low priority, especially in surgeries performed on the beating heart.^7^ In addition, many studies have suggested that revascularisation cannot be completed in patients who undergo surgery using this method.^8^

Early intervention and intra-operative evaluation of graft patency and quality are critical to surgical success. Detecting and solving technical problems during the intra-operative period is particularly effective in reducing the early occlusion of saphenous vein grafts. Tensions due to insufficient graft length and technical deficiencies in anastomosis can be effective in early graft occlusions.^9^,^10^

Although different methods have been developed to measure anastomosis quality (intra-operative angiography, thermal angiography, electromagnetic graft flow measurement), several studies have suggested that TTFM is more useful due to its easy application and lower cost.^11^-^13^ In line with these studies, we used the TTFM method to evaluate the quality of grafts in different surgical methods that were performed at our clinic.

In the recent study by Kaya et al. that evaluated 1 240 patients and 3 596 graft measurements at our clinic, TTFM proved to be quite effective at detecting peri-operative graft failure.^14^ In another study at our clinic that compared two groups with and without TTFMs, mortality, peri-operative and postoperative myocardial infarction, additional intervention when necessary, and the need for intra-aortic balloon pump support were found to be lower in the group with TTFMs.^15^

The data obtained with this method are presented as the PI, DF and mean flow. Although these cannot individually be used to sufficiently evaluate the quality of anastomosis, low graft flow is interpreted to represent an error in anastomosis. Some studies have indicated that mean flow values are not directly correlated with clinical outcome and graft patency in the long term.^16^,^17^

Despite the present study’s use of three different techniques, we did not detect any significant differences between the mean flow values of the grafts. Many studies have observed that comparisons that only involve the mean flow value are insufficient. It is possible to obtain high graft flow with a stenotic anastomosis. The study by Jaber et al. illustrated that there was no serious deterioration in the mean flow value when stenosis of the anastomoses remained below 75%.^18^

The PI is accepted as the most important parameter for determining the quality of anastomosis. In a study based on these values that compared on-pump with off-pump surgery, the results were in favour of on-pump surgery.^19^ In the present study, the PI values in group 1 were significantly lower than in the other groups. However, there were no significant differences between the groups in terms of the number of patients with PI values that required intervention. In other words, the mean PI values in the three groups were within acceptable limits.

Because the basis of our study was to detect and compare intra-operative measurements and additional intra-operative surgical interventions, if necessary, no comment was made on the long-term results of the grafts. Nonetheless, we believe that intra-operative comparisons of these data can prove highly useful in comparing anastomosis quality between surgical techniques.

Studies in the literature have indicated that acidosis^20^ and related coronary vasodilation^21^ develop in coronary systems during off-pump surgery. However, in the current study, a significant difference was detected in only the PI values of group 1 among the TTFMs in all three groups that had undergone operations with on-pump and off-pump surgical techniques. All groups were similar in the number of patients who presented PI values that were below the acceptable level (PI < 5). We believe that the absence of any significant differences between the TTFMs of patients undergoing on-pump operations through induced cardiac arrest, patients undergoing on-pump operations without induced cardiac arrest, and patients undergoing operations on the off-pump beating heart were due to the sufficient clinical experience involved in all three methods.

Nevertheless, the presence of lower PI values in the patients who underwent surgery through induced cardiac arrest may suggest that more ideal conditions can be provided for anastomosis with this method. Although no statistically significant differences were identified, lower anastomosis-related technical problems in patients who underwent on-pump surgery without induced cardiac arrest may indicate that surgeons felt safer with this method than with off-pump surgeries.

Th study by Kieser et al. showed that graft revisions are not always required in patients with a measured PI value of ≥ 5.22 In these patients, the PI value can be reduced to less than 5 with simple interventions, including corrections of the position of the graft. A PI value of ≥ 5 indicates that a graft has been revised. Of the patients with a PI value of ≥ 5, four showed sufficient improvement in their PI values with position changes, and three showed sufficient improvement with local papaverine administration but no graft revisions. As in the results of the study by Kieser et al., mechanical problems were eliminated, and the flows were improved prior to graft revision in cases of high PI values and in accordance with our data.^22^

Many factors affect graft flow, such as graft length and diameter, structure of the native vessel, mean arterial pressure, heart rate, competitive flow with the native vessel, and viscosity of the blood. As in the study by Kieser et al., because all conditions could not be standardised, we performed TTFMs after all the patients’ anastomoses were surgically completed and haemodynamic stabilisation had been achieved.^22^

Although arterial grafts used in coronary bypass surgery can remain in patients for longer periods of time than venous grafts, venous grafts are considered to provide better flow when the myocardium requires high levels of blood.^23^ In our study, we compared all grafts with the same grafts in the other groups. Therefore, the flow differences between arterial and venous grafts did not affect our results.

## Conclusion

The quality of anastomosis is the most important factor in coronary artery bypass surgery. Assessing anastomosis quality is an increasingly popular method for evaluating flow in grafts with TTFM and for performing additional surgical interventions based on these values. Studies that compare classical on-pump surgery, on-pump surgery without cardiac arrest, and off-pump surgery, which surgeons avoid since it may reduce anastomosis quality, are quite important. Because the results were similar for the three different methods used by the same surgical team, it is probable that all three methods can safely be used in the presence of sufficient surgical experience.
